# PI3K/Akt/mTOR Pathway and Its Role in Cancer Therapeutics: Are We Making Headway?

**DOI:** 10.3389/fonc.2022.819128

**Published:** 2022-03-24

**Authors:** Yan Peng, Yuanyuan Wang, Cheng Zhou, Wuxuan Mei, Changchun Zeng

**Affiliations:** ^1^ Department of Obstetrics, Longhua District Central Hospital, Shenzhen, China; ^2^ Xianning Medical College, Hubei University of Science and Technology, Xianning, China; ^3^ Department of Medical Laboratory, Shenzhen Longhua District Central Hospital, Guangdong Medical University, Shenzhen, China

**Keywords:** PI3K/Akt/mTOR pathway, targeted therapy, precision medicine, cancer, oncogenic alterations

## Abstract

Cancer is a severe public health issue that is a leading cause of mortality globally. It is also an impediment to improving life expectancy worldwide. Furthermore, the global burden of cancer incidence and death is continuously growing. Current therapeutic options are insufficient for patients, and tumor complexity and heterogeneity necessitate customized medicine or targeted therapy. It is critical to identify potential cancer therapeutic targets. Aberrant activation of the PI3K/AKT/mTOR pathway has a significant role in carcinogenesis. This review summarized oncogenic PI3K/Akt/mTOR pathway alterations in cancer and various cancer hallmarks associated with the PI3K/AKT/mTOR pathway, such as cell proliferation, autophagy, apoptosis, angiogenesis, epithelial-to-mesenchymal transition (EMT), and chemoresistance. Importantly, this review provided recent advances in PI3K/AKT/mTOR inhibitor research. Overall, an in-depth understanding of the association between the PI3K/AKT/mTOR pathway and tumorigenesis and the development of therapies targeting the PI3K/AKT/mTOR pathway will help make clinical decisions.

## Introduction

The mammalian target of rapamycin complex 1 (mTORC1) and the mammalian target of rapamycin complex 2 (mTORC2) are two distinct complexes formed by the mTOR. Growth factors, rapamycin, insulin, phosphatidic acid, certain amino acids, and oxidative stress affect the activity of mTOCR1, which is comprised of mTOR, Raptor, MLST8, PRAS40, and DEPTOR. The most classical targets downstream of mTOCR1 are S6K and 4EBP1, which play critical roles in protein synthesis, nutritional response, and tumor development. mTORC2 is composed of mTOR, RICTOR, mLST8, PROTOR1/2, DEPTOR, and mSIN1. mTORC2 interacts with PDK1 to activate AKT *via* phosphorylating it. Moreover, mTORC2 plays a critical role in the actin cytoskeleton, cell cycle, and survival (1–5). Receptor tyrosine kinases (RTKs), alterations in PIK3CA and its effectors, reduced PTEN expression, and other events contribute to oncogenic stimulation of the PI3K/Akt/mTOR pathway (6–8). In this review, we mainly described the genetic alterations of the PI3K/Akt/mTOR pathway. The mutations and amplification of PIK3CA are the most occurring events in cancer, and abnormal PI3K activity is a transforming event in the disease process (9). Alteration in AKT can cause an abnormal increase in the phosphorylated level of Akt in cancer cells (10). PTEN is another component of the PI3K/Akt signaling pathway, and its dysregulation can enhance cell growth, proliferation, and survival. Loss of heterozygosity (LOH), mutations, promoter methylation, and post-translational inhibition of PTEN are events in tumors and are involved in the pathogenesis of tumors (11, 12).

**Figure 1:**
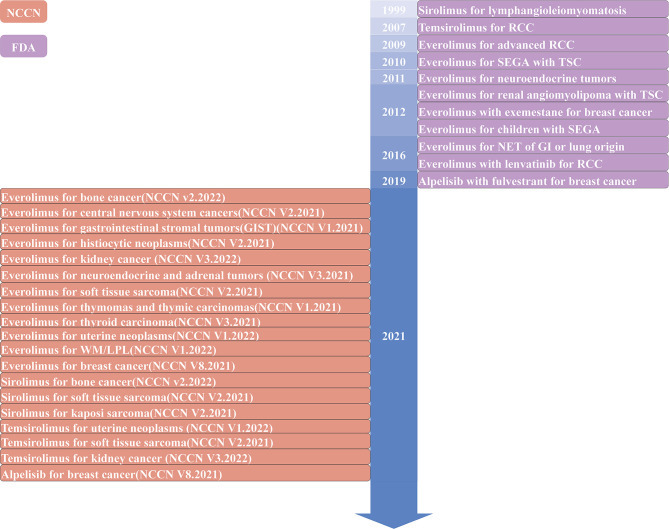
Timeline of The Food and Drug Administration (FDA)-approved or the National Comprehensive Cancer Network (NCCN) recommended inhibitors targeting the PI3K/AKT/mTOR pathway in solid tumors. WM, Waldenstrom macroglobulinemia; LPL, lymphoplasmacytic lymphoma; RCC, renal cell carcinoma; SEGA, subependymal giant cell astrocytoma; TSC, tuberous sclerosis complex; NET, neuroendocrine tumor; GI, gastrointestinal.

In this era of precision medicine, there are some advances in tailored, targeted therapies that inhibit specific pathways, potentially halting the evolution and spread of cancer. Understanding the abnormal expression of cancer pathway genes that play a vital role in cancer genesis and progression will contribute to cancer therapies. Based on current cancer genomic investigations, several critical cancer pathways are abnormally regulated (13). The PI3K/AKT/mTOR signaling pathway has been described as one of the most commonly disrupted pathways in cancer, making it an attractive candidate for therapeutic intervention. The PI3K/AKT/mTOR pathway is crucial for cell motility, growth, survival, and metabolism in cancer (14, 15). The current commercially accessible targeted inhibitors for cancer patients with abnormal activation of the PI3K/AKT/mTOR pathway include everolimus (mTOR inhibitor), sirolimus (mTOR inhibitor), temsirolimus (mTOR inhibitor), alpelisib (PI3K inhibitor), duvelisib (PI3K inhibitor), copanlisib (PI3K inhibitor), idelalisib (PI3K inhibitor), umbralisib (PI3K inhibitor). Phase III clinical trials of AKT inhibitors, such as capivasertib and ipatasertib, have been proceeded in cancer (**Figure 1** and **Table 1**) (6, 16, 17). Preclinical and clinical trials have shown encouraging outcomes for these targeted medicines. However, resistance to these medications is a drawback to their clinical usage. The rising prevalence of cancer necessitates the development of increasingly effective targeted medicines. Additionally, drugs targeting the PI3K/AKT/mTOR pathway in combination with chemotherapy drugs or other targeted drugs can inhibit tumor development (18). This review highlights the importance of the PI3K/AKT/mTOR pathway in cancer genesis and progression and summarizes inhibitors of this axis for cancer prevention and treatment.

**Table 1:** PI3K/AKT/mTOR pathway inhibitors.

Inhibitors	Targets	FDA-approved status	Clinical trial	Condition
Piqray (alpelisib) 2019 year	PI3Kα	Yes	NCT02437318(Phase III)	In combination with fulvestrant for postmenopausal women, and men, with HR-positive, HER2-negative, PIK3CA-mutated, advanced or metastatic breast cancer.
Copiktra (duvelisib) 2018 year	PI3Kδ, PI3Kγ	Yes	NCT02004522(Phase III);NCT02204982(Phase III)	Adult patients with relapsed or refractory CLL or SLL or FL after at least two prior therapies.
Aliqopa (copanlisib) 2017 year	PI3Kδ, PI3Kα	Yes	NCT01660451(Phase II)	Copanlisib for the treatment of adult patients with relapsed FL.
Zydelig (idelalisib) 2014 year	PI3Kδ	Yes	NCT01539512(Phase III)	Chronic lymphocytic leukemia, relapsed follicular B-cell non-Hodgkin lymphoma, and relapsed small lymphocytic lymphoma.
Ukoniq (umbralisib) 2021year	PI3Kδ, CK1ε	Yes	NCT02793583 (Phase II/III)	MZL and FL.
Sonolisib (PX-866) 2015 year	PI3Kα, PI3Kγ, PI3Kδ	No	NCT01259869(Phase II)	Glioblastoma multiforme at the time of first relapse or progression.
Buparlisib (NVP-BKM120) 2018 year	PI3Kα, PI3Kβ, PI3Kγ, PI3Kδ	No	NCT01633060(Phase III)	In combination fulvestrant, in postmenopausal women with HR-positive HER2-negative aromatase inhibitor-treated, locally advanced or metastatic breast cancer who progressed on or after mTOR inhibitor-based treatment.
Afinitor (everolimus) 2009 year	mTORC1, mTORC2	Yes	NCT00863655(Phase III); NCT00510068(Phase III); NCT01524783(Phase III); NCT00412061(Phase III); NCT00410124(Phase III)	Advanced HR+, HER2- breast cancer; PNET; progressive NET of gastrointestinal or lung origin; advanced renal cell carcinoma; SEGA and renal angiomyolipomas associated with tuberous sclerosis.
Rapamune (Sirolimus) 1999 year	mTORC1, mTORC2	Yes	NCT00414648(Phase III)	The prophylaxis of organ rejection in patients aged 13 years or older receiving renal transplants, and LAM.
Torisel (temsirolimus) 2007year	mTORC1, mTORC2	Yes	NCT00065468(Phase III)	Advanced renal cell carcinoma.
Capivasertib (AZD5363) 2020 year	AKT1, AKT2, AKT3	No	NCT03997123(Phase III)	Locally advanced or metastatic triple-negative breast cancer
Ipatasertib (GDC-0068) 2021 year	AKT1, AKT2, AKT3	No	NCT03072238(Phase III)	Ipatasertib plus abiraterone and prednisolone in metastatic castration-resistant prostate cancer
MK-2206 2020 year	AKT1, AKT2, AKT3	No	NCT01042379(Phase II)	HER2-positive and/or HR-negative breast cancer

HR: hormone receptor; HER2: epidermal growth factor receptor 2; CLL: chronic lymphocytic leukemia; SLL: small lymphocytic lymphoma; FL: follicular lymphoma; MZL: marginal zone lymphoma; PNET: progressive neuroendocrine tumors of pancreatic origin; NET: neuroendocrine tumors; SEGA: subependymal giant cell astrocytoma; LAM: lymphangioleiomyomatosis.

## Oncogenic PI3K/Akt/mTOR Pathway Alterations in Cancer

### Genetic Alterations in the PIK3CA Gene

**Figure 2:**
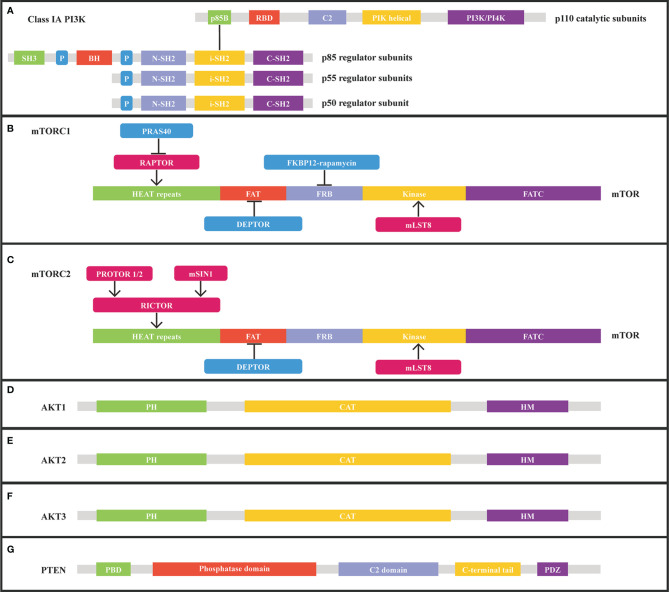
Structure of class IA PI3K, mTORC1, mTORC2, AKT1, AKT2, AKT3, and PTEN from Uniprot.org. **(A)** class IA PI3K consists of catalytic and regulatory subunits. **(B)** mTORC1 subunits and corresponding binding sites on mTOR. **(C)** mTORC2 subunits and corresponding binding sites on mTOR. **(D)** Structure of AKT1. **(E)** Structure of AKT2. **(F)** Structure of AKT3. **(G)** Structure of PTEN. RBD, Ras binding domain; P, proline-rich domain; BH, breakpoint cluster homology domain; C2, membrane-interacting domain; iSH2, inter-SH2 domain; FRB, FKBP12-rapamycin-binding; HEAT, Huntingtin/Elongation factor 3/a subunit of protein phosphatase2A/TOR1; mSIN1, mammalian stress-activated; PH, pleckstrin homology; CAT, catalytic domain; HM, hydrophobic motif.

The p110 α (p110α) subunit encoded by the PIK3CA gene is the most prevalent altered catalytic subunit of the phosphatidylinositol 3-kinase (PI3K) isoform in cancer. PI3K, comprised of a catalytic subunit (p110α) and a regulatory subunit (p85α), is a class of lipid kinases that participates in cellular functions, including cell proliferation, growth, differentiation, migration, and survival (**Figure 2A**). Numerous receptor tyrosine kinases, such as ERBB2, EGFR, MET, RET, and VEGFR, transform extracellular stimuli into intracellular signals and bind PI3K to the plasma membrane through scaffold proteins like IRS1 or by activation of RAS. After being stimulated, PI3K-110α transforms its lipid substrate phosphatidylinositol-4,5-bisphosphate (PIP2) to phosphatidylinositol- 3,4,5-bisphosphate (PIP3), triggering the AKT/mTOR pathway (19).

According to various studies, the mutation frequency of the PIK3CA gene ranges from 11% to 14% in cancer. PIK3CA activation mutations are identified in multiple tumor types, such as breast cancer, uterine corpus endometrial carcinoma, carcinoma of the uterine cervix, colorectal carcinoma, esophageal carcinoma, gallbladder carcinoma, non-small cell lung cancer, ovarian carcinoma, and gastric cancer (**Table 2**). 13% (1354/10336) of patients harbored PIK3CA mutations in MSK-IMPACT Clinical Sequencing Cohort, while 11% (1143/10194) of patients harbored PIK3CA mutations in China Pan-cancer Cohort (OrigiMed2020) (20, 22). The most common mutations in PIK3CA are in the helix domain (E542/E545) and the kinase domain (H1047) (22–24). PIK3CA mutations are prevalent in breast cancer, with 35.7% (2261/6338) of tumors carrying the mutations, most common in estrogen receptor-positive breast cancer. H1047R (35%), E545K (17%), E542K (11%), N345K (6%), and H1047L (3%) were the five mutations that accounted for 73% of all PIK3CA mutations (25). PIK3CA H1047R and H1047L are hotspot mutations within the PI3K/PI4K kinase domain of the Pik3ca protein. Moreover, the E545K and E542K mutations in the PIK helical domain of the Pik3ca protein are also hotspot mutations. Besides, PIK3CA N345K lies in the Pik3ca protein’s C2 PI3K-type domain. H1047R, H1047L, E545K, E542K, and N345K contribute to enhanced Akt and Mek1/2 phosphorylation, cell survival, and transformation (26, 27). PIK3CA amplification is a frequent genetic event in various cancers such as lung squamous cell carcinoma, cervical squamous cell carcinoma, esophageal adenocarcinoma, breast carcinoma, and is usually mutually exclusive with PIK3CA mutations. Increased PIK3CA copy number is strongly associated with increased PIK3CA expression and PI3K activity in malignancies. In addition, breast cancer patients with high PIK3CA copy numbers have a worse prognosis (22–24, 28).

**Table 2:** Genetic alterations of the PIK3CA, mTOR, PTEN, AKT1, AKT2, and AKT3 genes in human cancers.

PIK3CA			
Cancer Type	Number of Cases	Mutation Frequency	Amplification Frequency
Endometrial Carcinoma	586	44.54%	4.10%
Cervical Squamous Cell Carcinoma	251	22.71%	11.55%
Invasive Breast Carcinoma	1084	30.72%	1.85%
Head and Neck Squamous Cell Carcinoma	523	13.58%	11.85%
Colorectal Adenocarcinoma	594	24.75%	–
Bladder Urothelial Carcinoma	411	20.44%	2.68%
Non-Small Cell Lung Cancer	1053	5.41%	15.95%
Ovarian Epithelial Tumor	584	0.86%	19.35%
Esophagogastric Adenocarcinoma	514	14.20%	5.06%
Diffuse Glioma	513	8.19%	0.78%
Glioblastoma	592	6.25%	2.53%
Melanoma	444	4.73%	0.45%
Prostate Adenocarcinoma	494	2.02%	2.23%
mTOR			
Cancer Type	Number of Cases	Mutation Frequency	Amplification Frequency
Melanoma	444	11.94%	0.90%
Endometrial Carcinoma	586	10.58%	0.85%
Esophagogastric Adenocarcinoma	514	6.42%	1.75%
Colorectal Adenocarcinoma	594	6.73%	–
Renal Clear Cell Carcinoma	511	6.07%	–
Non-Small Cell Lung Cancer	1053	4.18%	0.19%
PTEN			
Cancer Type	Number of Cases	Mutation Frequency	Delation Frequency
Endometrial Carcinoma	586	58.02%	2.56%
Glioblastoma	592	22.13%	8.95%
Prostate Adenocarcinoma	494	2.63%	15.59%
Melanoma	444	9.46%	5.86%
Cervical Squamous Cell Carcinoma	251	7.57%	4.78%
Esophagogastric Adenocarcinoma	514	6.03%	4.28%
Invasive Breast Carcinoma	1084	5.17%	4.98%
Non-Small Cell Lung Cancer	1053	5.60%	4.75%
Sarcoma	255	2.35%	5.88%
Colorectal Adenocarcinoma	594	5.22%	2.36%
Bladder Urothelial Carcinoma	411	3.89%	3.16%
Ovarian Epithelial Tumor	584	1.20%	4.45%
Diffuse Glioma	513	4.68%	0.97%
Hepatocellular Carcinoma	369	1.90%	3.52%
Head and Neck Squamous Cell Carcinoma	523	2.29%	2.87%
AKT1			
Cancer Type	Number of Cases	Mutation Frequency	Amplification Frequency
Endometrial Carcinoma	586	3.24%	1.02%
Ovarian Epithelial Tumor	584	3.94%	0.17%
Invasive Breast Carcinoma	1084	2.49%	1.11%
Melanoma	444	2.70%	–
Cervical Squamous Cell Carcinoma	251	1.59%	1.20%
Non-Small Cell Lung Cancer	1053	0.85%	1.71%
AKT2			
Cancer Type	Number of Cases	Mutation Frequency	Amplification Frequency
Pancreatic Adenocarcinoma	184	0.54%	7.07%
Endometrial Carcinoma	586	3.58%	3.57%
Ovarian Epithelial Tumor	584	0.17%	5.31%
Cervical Squamous Cell Carcinoma	251	1.99%	3.59%
Sarcoma	255	0.39%	3.53%
Non-Small Cell Lung Cancer	1053	1.14%	3.32%
Esophagogastric Adenocarcinoma	514	2.14%	1.17%
Bladder Urothelial Carcinoma	411	0.97%	2.43%
AKT3			
Cancer Type	Number of Cases	Mutation Frequency	Amplification Frequency
Invasive Breast Carcinoma	1084	0.74%	9.78%
Endometrial Carcinoma	586	5.29%	2.73%
Hepatocellular Carcinoma	369	0.27%	5.96%
Melanoma	444	2.48%	2.93%
Ovarian Epithelial Tumor	584	5.65%	–
Non-Small Cell Lung Cancer	1053	1.14%	3.51%
Esophagogastric Adenocarcinoma	514	1.75%	1.36%
Pancreatic Adenocarcinoma	184	0.54%	2.17%
Colorectal Adenocarcinoma	594	2.19%	0.51%

The Cancer Genome Atlas (TCGA) PanCancer Atlas Studies included 32 studies selected (10967 samples) (20, 21).

### Genetic Alterations in the mTOR Gene

The mTOR protein, which is encoded by the mTOR gene, belongs to a serine-threonine kinase that controls cell responses to stressors such as growth factors, nutrient deprivation, and DNA damage and regulates tumor growth, survival, and metabolic signaling (**Figures 2B, C**). mTOR activation mutations enhance the kinase activity of mTOR, resulting in the overactivation of downstream pro-proliferative pathways. MTOR mutations are common in malignant tumors, such as endometrial carcinoma, melanoma, esophagogastric adenocarcinoma, colorectal adenocarcinoma, renal cell carcinoma, and bladder cancer (**Table 2**) (29). In MSK-IMPACT Clinical Sequencing Cohort, MTOR is mutated at a rate of 3% (329/10336) in metastatic cancer, which is also observed at the similar rate (2.9%, 292/10194) observed in China Pan-cancer Cohort (OrigiMed2020) (20, 22). Nonsynonymous mTOR mutations are present in 10.4% (N=412) of melanoma patients and are associated with a poor prognosis (30). Besides, 6% of clear-cell renal cell carcinoma patients with mTOR mutation were identified (31). The predicted mutation incidence was 3% (N=8630) for mTOR in head and neck cancer (32). The mTOR missense mutations are found in a wide variety of malignancies, most notably in roughly 7.5% of lung adenocarcinomas, 6% of clear cell renal cell carcinomas, 5% of endometrial carcinomas, and 4% of colorectal carcinomas (33). The most prevalent alterations of MTOR in malignant tumors are E1799K, S2215F, and amplification (29). Mutations in key regions such as HEAT repeat, FAT domain, and kinase domain make the mTOR gene highly tumorigenic. Moreover, mTOR W1456R, M938T, V2284M, T2294I, V2291I, P2273S, G1479N, and E2288K mutants dramatically elevated the activity of protein kinase. Besides, the mTOR/p70S6K pathway was significantly increased in the W1456R, P2273S, and E2288K mutants. In addition, the W1456R, P2273S, and E2288K mutants affected the mTOR/p70S6K and Akt pathways (34).

### Genetic Alterations in the AKT Gene

AKT1, AKT2, and AKT3, as members of the AGC kinase family, are serine/threonine protein kinases and downstream effectors of the PI3K signaling pathway (**Figures 2D–F**). Following PI3K activation, cytosolic AKT1 is transported to the membrane where it interacts with PIP3 (PtdIns3,4,5-P3), resulting in AKT1 phosphorylation and activation. AKT1 can stimulate a variety of downstream effectors, including GSK3, FOXO, and mTORC1, all of which are crucial for cell survival, growth, and metabolism. AKT1 can be negatively regulated as a result of PTEN phosphatase activity inhibiting PI3K. Besides, activation of the PI3K pathway or inactivation of PTEN can cause AKT1 activation in cancers. AKT1 activation mutations and AKT1 infrequent amplification enable AKT1 activation independent of phosphoinositide (14, 35, 36). The activating mutations of AKT2 and AKT3 induce disruption of intramolecular pleckstrin homology domain (PH) and kinase domain (KD) interactions, resulting in AKT oncogenic activation (37). AKT1, AKT2, AKT3 aberrations were identified in 1.8% (183/10336), 1.6% (163/10336), and 1.4% (149/10336) of patients in the MSK-IMPACT Clinical Sequencing Cohort, respectively. While AKT1, AKT2, AKT3 aberrations were identified in 1.4% (138/10194), 2% (206/10194), and 1.2% (122/10194) of patients in China Pan-cancer Cohort (OrigiMed2020) (20, 22). AKT1 E17K is a hotspot mutation, the most frequent AKT1 mutation in breast cancer, and a highly recurrent AKT1 mutation in many other cancer types. 6.3% (N=619) of breast cancer patients carry the AKT1 E17K mutation, associated with increased mortality (38). AKT1 E17K mutation boosts the binding of Akt1 to the phosphatidylinositol-3,4,5-trisphosphate (PIP3) ligand, which facilitates Akt transport from the cytoplasm to the cell membrane, and further stimulates Akt phosphorylation on the cell membrane. Activated AKT relocates in the cytoplasm, nucleus, or other intracellular locations phosphorylates a wide variety of substrate proteins and consequently modulates cell activity. Besides, AKT1 E17K mutation accelerates cell migration and resistance to chemotherapeutic treatments in luminal breast cancer cells (39, 40). The oncogene AKT2 is triggered by amplification or overexpression in a variety of malignant tumors, thus facilitating tumor invasion and metastasis (41, 42). More prevalent amplification of the oncogene AKT3 has been detected in many cancers, such as breast carcinoma, endometrial carcinoma, melanoma, ovarian epithelial tumor, cholangiocarcinoma, and non-small cell lung cancer (**Table 2**) (22, 23).

### Genetic Alterations in the PTEN Gene

PTEN is a tumor suppressor gene that can negatively regulate the PI3K/AKT/mTOR pathway and is one of the most common mutated genes in cancer (**Figure 2G** and **Table 2**). PTEN functions as a phosphatase on the cell membrane, converting phosphatidylinositol (3–5)-triphosphate (PIP3) to phosphatidylinositol (4, 5)-diphosphate (PIP2). PTEN dysfunction caused by inactivation mutations, homozygous deletions, loss of heterozygosity (LOH), or epigenetic modifications accumulates PIP3 and activates catabolic downstream AKT/mTOR signaling, thereby stimulating cell proliferation and survival. In addition, nuclear PTEN can modulate RAD51 expression, which is tightly associated with homologous recombination (HR) and DNA double-strand breaks (DSBs). Furthermore, PTEN deficiency may also result in increased genomic instability, allowing for the accumulation of deleterious mutations (43). Nedd8 interacts with PTEN at high glucose levels, inducing PTEN neddylation and resulting in nuclear import of PTEN without impairing PTEN stability. Neddylated PTEN mainly aggregates in the nucleus and dephosphorylates the fatty acid synthase (FASN), suppresses FASN ubiquitylation and degradation through TRIM21, and subsequently enhances fatty acid synthesis. Besides, PTEN neddylation was closely associated with tumor development and a worse prognosis in breast cancer (44). Germline PTEN mutations are found in approximately 80% of patients with the cancer predisposition syndrome Cowden, which is associated with a high incidence of breast and thyroid cancer events (45, 46). The PTEN mutation is one of the most prevalent cancer mutations, frequently found in endometrial carcinoma, glioblastoma, and prostate adenocarcinoma. PTEN mutations occurred in 9% (888/10336) of patients in the MSK-IMPACT Clinical Sequencing Cohort, while PTEN was altered in only 5% (534/10194) of patients in China Pan-cancer Cohort (OrigiMed2020) (20, 22). PTEN mutations or deletions are detected in 45% of endometrial cancer and are more frequent in endometrioid endometrial cancer than in other histological subtypes (47). PTEN mutations were found in 29.0% (N=303) of glioblastoma patients, and PTEN deletions were identified in 39.6% (N=260) of glioblastoma cases. 13.5% (N=260) of cases had concomitant PTEN mutation and deletions (48). PTEN mutations and/or deletions are identified in 30% of prostate cancer cases. PTEN silencing lowers H3K27me3 and H3K27Ac enrichment in the Nkx3.1 promoter region and promotes alterations in DNA CpG methylation and transcriptome gene expression, associated with various inflammatory and immunological pathways in the development of prostate cancer (49). PTEN Y240 phosphorylation mediated by FGFR2 prevents cells from DNA damage *via* enabling DNA repair. PTEN Y240 phosphorylation facilitates homologous recombination (HR)-mediated DNA double-strand break (DSB) repair through enhancing RAD51 filament synthesis or stabilization. PTEN Y240 phosphorylation interacts with chromatin and recruits RAD51 to facilitate DNA repair. Inhibiting Y240 phosphorylation can make glioblastoma more sensitive to ionizing radiation (IR), thus extending glioblastoma survival time (50).

## The PI3K/AKT/mTOR Pathway and Different Cellular Processes in Cancer

### Cell Proliferation

PI3K/Akt/mTOR signaling pathway involved in cell survival, growth, and proliferation is the commonly activated signaling pathway in human cancers. Dysregulated mTOR activation is a frequent observation in cancer and represents a process in cancerogenesis. mTOR interacts with other proteins and is a component of two protein complexes, mTOR complex 1 and mTOR complex 2, that control various cellular activities. Both mTORC1 and mTORC2 contain subunits that mediate different but overlapping activities. mTORC1 is triggered by a variety of nutrients and can be stimulated by PI3K signaling. mTORC1 is an upstream regulator, whereas mTORC2 is a downstream effector of Akt. Akt is an essential substrate of mTORC2, which is often shown to be overactive in malignancies. Akt accumulates signals from the PI3K/mTORC2 and PI3K/PDK1 to enhance cell survival, growth, and proliferation (51). mTORC1 regulates the phosphorylation of downstream translation effectors such as the ribosomal protein S6 kinase B1 (S6K1) and the eukaryotic translation initiation factor 4E (eIF4E)-binding protein 1 (4E-BP1) to control cell growth and proliferation. mTORC2 controls cell survival and proliferation *via* phosphorylating Akt Ser473. Small molecules, such as hormones and growth factors, can activate Akt, mTORC2, and then mTORC1 through an Akt-dependent phosphorylation pathway. Nutrients can activate Akt, mTORC2, and directly stimulate mTORC1 through an Akt-independent phosphorylation pathway. Glycogen synthase kinase-3 beta (GSK3) is a significant Akt substrate, and its deactivation is induced by phosphorylation. GSK3 promotes cell proliferation by modulating the stability and production of proteins involved in the G1/S cell cycle phase transition, such as cyclin D1. FKBP4 enhances cell proliferation in breast cancer by increasing Akt phosphorylation at Ser473 and Thr308 through PI3K/PDK1 and mTORC2 (52). GSK3 collaborates with mTORC1 by increasing p70S6K1 activity by phosphorylating at Ser371 inside the p70S6K1 turn motif, which enhances mTORC1- mediated Thr389 phosphorylation. GSK3 may serve as an inducer of malignant cell growth and survival. Phosphorylation of some GSK3 substrates is critical for cell proliferation or survival and produces a phosphorylated protein recognized by E3 ubiquitin ligase, causing the phosphorylated protein to be degraded by the proteasome. After Rictor is phosphorylated by GSK3 and interacts with FBXW-7, it is degraded through a ubiquitination/proteasome-dependent pathway. When PI3K/Akt signaling inactivates GSK3, Rictor expression, and mTORC2 assembly increase, boosting mTORC2 activity (53). Rictor functions as an upstream kinase for many members of the AGC (cAMP-dependent, cGMP-dependent, and protein kinase C) protein family, such as Akt, SGK, and PKC, and is a critical component of mTORC2. Stimulation of Rictor/mTORC2 affects the structure of actin and enhances cell proliferation by phosphorylating the substrates. When used in conjunction with rapamycin, Rictor down-regulation significantly decreased cell proliferation, increased cell cycle arrest, and induced apoptosis by inhibiting the AktSer473 feedback phosphorylation (54). Activation of the mTORC2 subunit p-AKT (Ser473) and RICTOR stimulate the esophageal squamous cell carcinoma. Inhibiting RICTOR may increase esophageal squamous cell carcinoma cell sensitivity to PP242 (a pan-mTOR inhibitor) as well as RAD001 (a mTORC1 inhibitor) (55).

### Autophagy

Autophagy is a critical homeostatic cellular recycling process that degrades damaged or dysfunctional cellular proteins and organelles. As a result of the dysregulation of the PI3K/Akt/mTOR pathway, autophagy can be triggered in malignancies, allowing them to adapt to low-nutrient environments and proliferate (56). mTOR is a regulator that inhibits autophagy, and anticancer therapies that disrupt the PI3K/Akt/mTOR pathway promote autophagy. mTORC1 inhibits catabolism *via* suppressing autophagy and lysosome formation, which are two processes critical for lysosome-dependent macromolecule degradation. mTORC1 inhibits autophagy and lysosomal degradation *via* phosphorylating ULK1, a critical autophagy modulator, and TFEB, a modulator of lysosomal gene expression. The activity of mTORC1 can be modulated by energy levels, nutrient status, and hypoxic settings *via* the AMPK/TSC pathway, which affects autophagy. The mTORC1 downstream effectors’ elongation factor 4E-BP1 and p70S6 kinase control protein synthesis. Stimulated mTORC1 phosphorylates the autophagy protein complex (ULK1/2) to suppress the downstream autophagy cascade. AMPK activated by AMP or LKB1 can promote autophagy by inhibiting the activity of mTORC1 through phosphorylation of TSC1/2. The intracellular flow of essential amino acids may limit autophagy by stimulating mTORC1. Additionally, MEK/ERK signaling promotes starvation-induced autophagy and ROS-dependent ERK activation boosts autophagy and induces cell death (57). PI3KCI stimulates Akt, which can attenuate the inhibitory impact of the TSC1/2 heterodimer on Rheb, thereby activating mTORC1 and inhibiting autophagy. Akt is triggered by mTORC2, which further inhibits autophagy. Moreover, PTEN promotes autophagy by suppressing the production of PIP3, which in turn triggers the PI3K/Akt/mTOR signaling. GTP-Ras suppresses autophagy *via* activating PI3KCI and the RAF/MEK/ERK pathway (58). The cyclin–CDK inhibitor CDKN1B (also known as p27Kip1) enhances starvation-induced autophagy *via* a mTORC1-dependent pathway. A portion of p27Kip1 is transported to lysosomes in amino acid-derived cells, where it cooperates with LAMTOR1, an essential component of the Ragulator complex, to stimulate mTORC1. When p27Kip1 binds to LAMTOR1, regulatory assembly and mTORC1 stimulation are inhibited, thereby facilitating autophagy. In p27−/− cells, elevated mTORC1 activity contributes to cytoplasmic retention of TFEB, impaired lysosomal function, and decreased autophagy flux, ultimately improving cell survival (59). Gαq, a component of the mTOR/Raptor/p62 complex, regulates autophagy by promoting the assembly of the active mTORC1 complex through PB1-mediated Gαq/p62 interaction in the presence of nutrients (60).

### Apoptosis

Apoptosis is a type of programmed cell death that enables the body to clear abnormal or unneeded cells in an orderly manner. Caspases are critical to the apoptotic mechanism as the initiators and executors of apoptosis, which can be activated by the extrinsic death receptor pathway, the intrinsic mitochondrial pathway, and the intrinsic endoplasmic reticulum pathway. The extrinsic death receptor pathway begins with the binding of death ligands (TNF and FasL) to death receptors (TNFR1 and Fas). The binding of the death ligand to the death receptor promotes the formation of the death-inducing signaling complex (DISC), a ligand-receptor-conjugating protein complex that further leads to the assembly and activation of caspase 8. The activated caspase 8 serves as an initiator caspase, which triggers apoptosis by cleaving other downstream caspases. Moreover, internal stimuli such as genetic damage, hypoxia, excessive cytosolic Ca2+ concentrations, and severe oxidative stress may cause elevated mitochondrial permeability and the production of pro-apoptotic substances such as cytochrome-c into the cytoplasm, stimulating the intrinsic mitochondrial pathway. Cytoplasmic release of cytochrome c activates caspase 3 *via* forming an apoptosome complex, composed of cytochrome c, Apaf-1, and caspase 9. The intrinsic mitochondrial pathway is tightly regulated by the Bcl-2 family proteins, mainly composed of pro-apoptotic proteins and anti-apoptotic proteins. Anti-apoptotic proteins such as Bcl-2, Bcl-XL, Bcl-W, BFL-1, and McL-1 regulate apoptosis by inhibiting the release of cytochrome c from mitochondria, while pro-apoptotic proteins such as Bax, Bak, Bad, Bcl-XS, Bid, Bik, Bim, and Hrk enhance the release of cytochrome c from mitochondria. Both extrinsic and intrinsic pathways are closely associated with various signaling proteins, such as NK-kB and p53-MDM2, and converge to caspases. Additionally, the intrinsic endoplasmic reticulum pathway is caspase 12-dependent and mitochondria-independent. Overall, the mechanisms of apoptosis evasion and carcinogenesis are mediated by the imbalance of Bcl-2 family proteins, reduced caspases expression, disrupted death receptor signaling pathway, p53 defects/mutations, and overexpression of inhibitor of apoptosis proteins (IAPs) such as BIRC1 (NAIP), BIRC2 (c-IAP1), BIRC3 (c-IAP2), BIRC4 (XIAP), BIRC5 (Survivin), BIRC6 (Apollon/BRUCE), BIRC7 (Livin/MLIAP) and BIRC8 (ILP2) (61, 62). mTOR inhibitors can rapidly inhibit 4E-BP1 phosphorylation *via* DR5/FADD/Caspase-8 axis and trigger the extrinsic apoptotic pathway in colorectal cancer cells. High dosages of mTOR inhibitors result in a significant reduction of 4E-BP1 phosphorylation and mTOR activity, all of which may be factors in ER stress, C/EBP homologous protein (CHOP), and death receptor 5 (DR5) and consequent cancer cell death. Besides, mTOR inhibitors have a substantial synergistic effect with tumor necrosis factor-related apoptosis-inducing ligand (TRAIL) and chemotherapy in inducing Fas-associated protein with death domain (FADD) and DR5-dependent apoptosis. Generally, mTOR inhibitors may have an anti-tumor effect *via* stimulating the extrinsic apoptotic pathway (63).

### Angiogenesis

Angiogenesis is the formation of new blood vessels, enabling oxygen and nutrients to be delivered to the body’s tissues. Angiogenesis is critical in the development of cancer, which requires the development of new blood vessels to grow and metastasize. Endogenous angiogenesis regulators mainly include growth factors, cytokines, proteases, protease inhibitors, trace elements, oncogenes, and endogenous modulators. A balance of activators such as vascular endothelial growth factor (VEGF), basic fibroblast growth factor (bFGF), platelet-derived endothelial cell growth factor (PD-ECGF), tumor necrosis factor (TNF)-α, angiogenin, transforming growth factor (TGF)-α, TGF-β, granulocyte colony-stimulating factor (G-CSF), placental growth factor (PGF), hepatocyte growth factor (HGF), interleukin-8 (IL-8), and epidermal growth factor (EGF) and inhibitors such as angiostatin, interferon, endostatin, platelet factor 4 (PF4), thrombospondin (TSP), and tissue inhibitors of metalloproteinases (TIMPs) regulates angiogenesis (64–66). mTOR, a crucial switch that regulates anabolism and catabolism of endothelial cells, is critical in angiogenesis. Shear stress and coordinated interactions between endothelial growth factors (VEGF, PDGF-B, ANG2, ANG1, bFGF, ephrin-B2, and TGF-beta superfamily), intracellular signaling molecules (NOTCH1 and COUP-TFII), and intercellular connections (VCAM1) contribute to tumor angiogenesis, which may activate the PI3K/Akt/mTOR pathway in cancer cells. Hypoxia stabilizes HIF-1α, which triggers tumor cells to produce more vascular endothelial growth factor (VEGF). Stimulation of the PI3K/AKT pathway in tumor cells may boost the production of VEGF *via* hypoxia-inducible factor 1 (HIF-1) dependent and independent pathways. Besides, the PI3K/AKT pathway also regulates other angiogenic factors such as angiopoietins and nitric oxide. Angiogenic tumor cells release more bFGF and VEGF than non-angiogenic tumor cells. Increased VEGF levels may enhance vascular permeability, resulting in leaky vessels, slow blood flow, and high interstitial pressure. The binding of VEGF to receptors stimulates the PI3K/AKT/mTOR pathway, which is critical for cell migration (67). Suppression of the mTOR pathway prevents VEGF-mediated angiogenesis and cell proliferation by decreasing VEGF production and secretion, and VEGFR2-mediated signaling. Rutacecarpine suppresses angiogenesis *via* targeting VEGFR2 and the Akt/mTOR/p70s6k signaling pathways regulated by VEGFR2 (68). Rapamycin inhibits angiogenesis and lymphangiogenesis in melanoma by inhibiting the expression of VEGF-A/VEGFR-2 and VEGF-C/VEGFR-3 (69). VEGF stimulation increased the expression of Sox7 and Sox17 in angiogenesis through the mTOR pathway. Additionally, Sox7 and Sox17 increased the expression of VEGFR2 in angiogenic vessels (70).

### Epithelial-to-Mesenchymal Transition

Epithelial-mesenchymal transition (EMT) refers to the transition of cells from epithelial to mesenchymal phenotypes, which results in functional alterations in cell migration and invasion. EMT is a vital step through which epithelial cells develop mesenchymal, fibroblast-like features characterized by decreased intercellular adhesion and enhanced motility. EMT-like processes play a significant role in tumor growth and malignant transformation, causing cancer cells to become more aggressive and spread (71, 72). During EMT, the expression of epithelial markers (E-cadherin) decreases, and the expression of mesenchymal markers (vimentin and fibronectin) increases. Snail, ZEB, and Twist regulate these markers by blocking the CDH1 gene encoding E-cadherin. Cumulative evidence indicates that the AKT/mTOR pathway is closely associated with the EMT process (73, 74). Phosphorylation and activation of EMT transcription factors are a result of AKT activation. Increased AKT activation causes decreased E-cadherin expression, partly attributable to the Snail accumulation in the nucleus. Besides, AKT may trigger Twist expression, which decreases E-cadherin expression and inhibits cell migration. Activation of NF-kB *via* AKT may cause the accumulation of ZEB-1, which acts as a repressor of E-cadherin expression. Growth factors, specific proteins, and aberrant tumor suppressors may stimulate AKT and regulate EMT transcription factor expression and EMT activation. Cancer cells experience dramatic remodeling of the actin cytoskeleton during the EMT process. mTORC2 modulates the actin cytoskeleton of the cell and EMT *via* regulating the phosphorylation status of Protein Kinase C (PKC) and activating Akt (75). TGF-receptors interact with the regulatory p85 subunit of PI3K, triggering the PI3K/AKT pathway. TGF-β has the potential to cause EMT *via* activating the mTOR pathway (76–79).

### Chemoresistance

The druggable metabolic vulnerability is mediated by mTORC1, which inhibits autophagy and enhances resistance to chemotherapy and targeted drugs (80–82). The mTOR pathway regulates FANCD2, which leads to cancer cells’ resistance to DNA double-strand breaks (83). The combination of adriamycin/cisplatin and mTOR inhibitor (torisel) blocked 4EBP-1 and p70S6K phosphorylation, elevated γH2AX expression indicative of DNA damage, and induced cell cycle arrest at G2/M and apoptosis, indicating a potential role of mTOR inhibitors in rebuilding chemosensitivity to adriamycin/cisplatin (84). FOXD1-AS1 facilitated the translation of FOXD1 protein *via* the eIF4G-eIF4E-eIF4A translational complex. Moreover, FOXD1-AS1 modulated 4E-BP1 phosphorylation and enhanced eIF4G-eIF4E interaction *via* triggering the PI3K/AKT/mTOR pathway. Overall, FOXD1-AS1 increases FOXD1 translation through PI3K/AKT/mTOR signaling, thereby exacerbating gastric cancer development and resistance to chemotherapy (85). Chemotherapy-resistant epithelial ovarian cancer cells exhibit EMT and increased expression of tumor stem cell markers due to stimulation of the PI3K/Akt/mTOR signaling pathway. In combination with cisplatin, BEZ235, an anti-PI3K/mTOR inhibitor, might be a viable therapeutic approach for epithelial ovarian cancer chemoresistance (86). Aurora-A expression was positively correlated with phosphorylated AKT/4E-BP1 expression in endometrial cancer tissues. Aurora-A triggers the AKT/mTOR pathway in endometrial cancer, which stimulates cell proliferation and causes chemoresistance, suggesting that Aurora-A inhibitor and AKT/mTOR inhibitor in combination with chemotherapy intervention may be a therapeutic strategy for Aurora-A overexpressed endometrial cancer (87).

## Recent Advances in PI3K/AKT/mTOR Inhibitor Research

**Figure 3:**
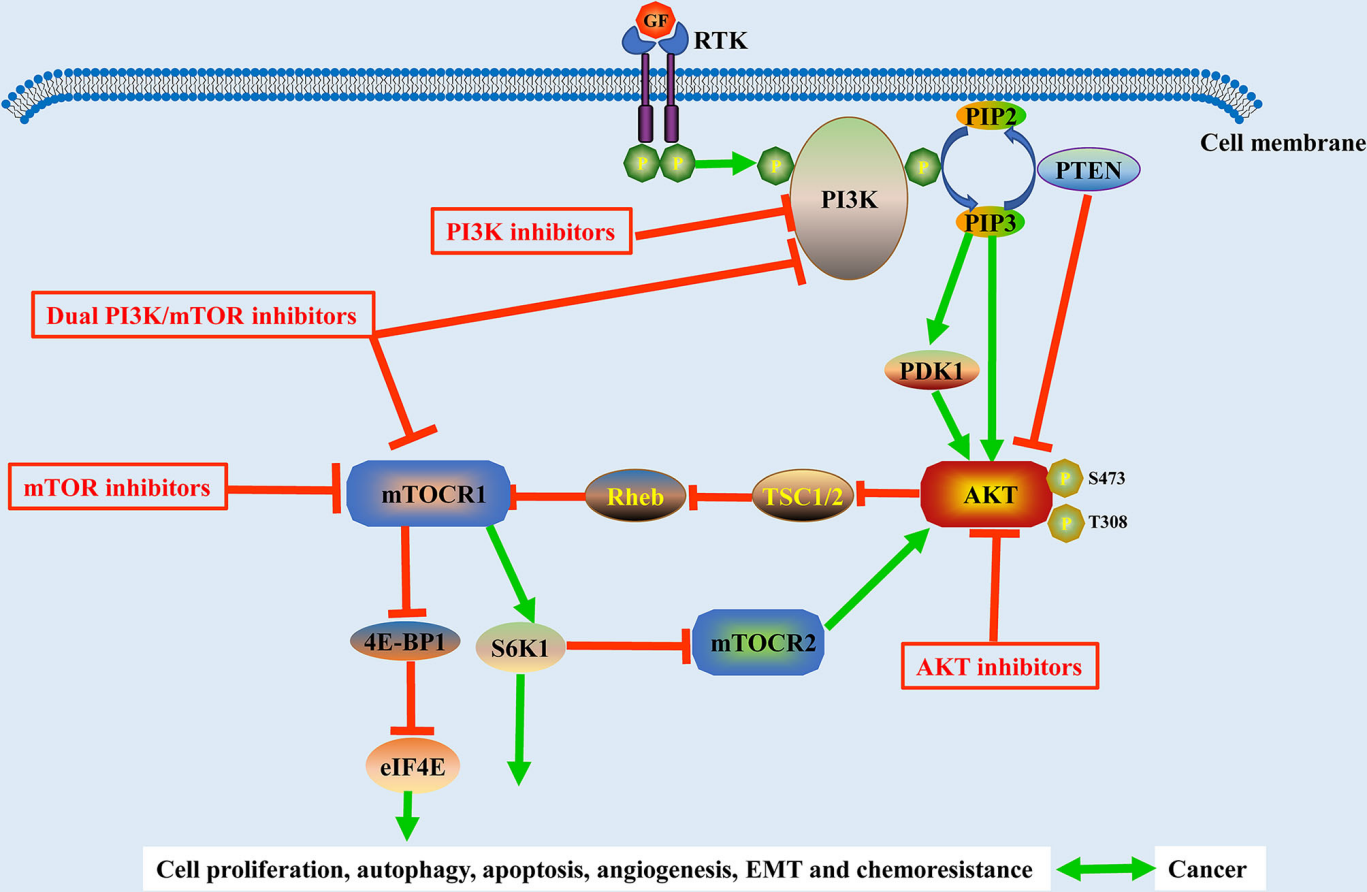
Schematic representation of the PI3K/Akt/mTOR pathway and its related inhibitors in solid tumors. The activation of the PI3K/Akt/mTOR pathway is associated with cell proliferation, autophagy, apoptosis, angiogenesis, EMT, and chemoresistance in solid tumors. GF, growth factor; EMT, epithelial-to-mesenchymal transition; RTK, tyrosine kinase receptor.

Because of the significant rise in the number of novel therapeutic drugs that target molecular pathways, targeted therapeutic agents are now more precise than traditional chemotherapy agents. The PI3K/AKT/mTOR pathway is critical for cell growth, proliferation, and survival, and it is one of the most frequently disrupted pathways in malignancies, making it a desirable target for treatment (**Figure 3**). In this review, we reviewed the research progress of PI3K/AKT/mTOR inhibitors and presented the representative PI3K/AKT/mTOR inhibitors in **Table 1**.

### PI3K Inhibitors

Phosphatidylinositol 3−kinases (PI3Ks) are a class of lipid kinases that control signaling and intracellular vesicular trafficking *via* phosphorylating intracellular inositol lipids. PI3Ks are classified into three groups based on their structural characteristic and substrate specificity. Class I PI3Ks synthesize 3-phosphoinositide lipids that activate signal transduction pathways directly. Class I PI3Ks are classified into subclasses IA and IB according to their regulatory mechanisms. Class IA PI3Ks are heterodimers with a catalytic subunit of p110 and a regulatory subunit of p85. The class IA catalytic isoforms p110α, p110β, and p110δ are encoded by the PIK3CA, PIK3CB, and PIK3CD genes, respectively. The class IB PI3K are the heterodimers with a catalytic subunit of p110γ and a regulatory subunit of p101 or p87. The class IB isoforms p110γ, p101, or p87 are encoded by the PIK3CG, PIK3R5, and PIK3R6 genes, respectively. The class I PI3Ks are frequently activated in malignant tumors, associated with translation, cell growth, glucose metabolism, cytoskeletal motility, cell survival, transformation. Cellular processes such as growth, cell migration, primary cilium function, glucose metabolism, cell survival, and angiogenesis are modulated by the Class II PI3Ks. The class II catalytic isoforms PI3KC2α, PI3KC2β, and PI3KC2γ are encoded by the PIK3C2A, PIK3C2B, and PIK3C2G genes, respectively. Autophagy, endosomal trafficking, and phagocytosis are dependent on the class III PI3K. The class III PI3Ks are the heterodimers with a catalytic subunit of VPS34 encoded by PIK3C3 and a regulatory and accessory subunit of VPS15 encoded by PIK3R4. Presently, several types of PI3K-specific inhibitors have been developed. PI3K inhibitors are divided into three categories according to their pharmacokinetic characteristics and capacity to interact with ATP-binding clefts: pan-PI3K inhibitors, isoform-selective PI3K inhibitors, and dual PI3K/mTOR inhibitors.

Pan-Class I inhibitors can block catalytic properties of p110 isoforms. The use of pan-PI3K inhibitors was restricted owing to the adverse pharmacological event caused by off-target effects, and on-target consequences of blocking all class I PI3K isoforms, independent of their role in carcinogenesis. PX-866, a derivative of Wortmannin, is a physiologically stable pan-PI3K inhibitor that targets the PI3K pathway and has shown improved antineoplastic activity and favorable pharmacokinetic properties in a variety of tumors (88). In glioblastoma, blockage of the PI3K pathway by PX-866 results in cell growth suppression and reduced stimulation of downstream pathways. PX-866 was generally well tolerated. However, it failed to achieve the efficacy endpoints. 21% of individuals with recurrent glioblastoma had sustained stable disease. However, there are no biomarkers that can distinguish participants (89). Buparlisib (NVP-BKM120) is a pan-PI3K inhibitor targeting all class I PI3K isoforms. Based on the safety profile of buparlisib with fulvestrant in the BELLE-3 trial, further studies on buparlisib with fulvestrant in postmenopausal, hormone-receptor-positive, HER2-negative, advanced breast cancer patients pretreated with endocrine treatment and mTOR inhibitors are not recommended. However, the efficacy of buparlisib with fulvestrant supports the use of PI3K inhibitors combined with endocrine therapy in individuals with PIK3CA mutations, indicating that PIK3CA mutations may be a biomarker of PI3K inhibitor efficacy (90). Buparlisib had limited single-agent efficacy in PI3K-activated recurrent glioblastoma. The lack of efficacy of buparlisib was attributed to inadequate blockage of the PI3K pathway, despite the drug’s substantial brain penetration. Further studies on PI3K inhibitors with more pathway blocking are needed (91). In the BERIL-1 study, patients with relapsed or metastatic head and neck squamous cell carcinoma treated with buparlisib in combination with paclitaxel had a median progression-free survival of 1.1 months longer than those treated with placebo plus paclitaxel (4.6 months *vs*. 3.5 months). Although the adverse events were considered manageable, patients treated with buparlisib plus paclitaxel had more grade 3 or 4 adverse events. Buparlisib plus paclitaxel seemed to be an effective second-line therapy for patients with platinum-pretreated recurrent or metastatic head and neck squamous cell carcinoma (92). In 2017, the Food and Drug Administration (FDA) granted approval to copanlisib (a pan-PI3K inhibitor) based on results from the CHRONOS-1 trial for the treatment of adult patients with recurrent follicular lymphoma who have undergone at least two previous systemic therapies (93). In addition to PX-866, buparlisib, and copanlisib, other pan-PI3K inhibitors include CH5132799, pilaralisib, ZSTK474, sonolisib, pictilisib, B591, TG-100-115, and RIDR-PI-103. Further clinical trials are needed to evaluate the efficacy of pan-PI3K inhibitors in solid tumors. The wide activity of pan-PI3K inhibitor may increase the risk of adverse effects and toxicity.

Isoform-selective PI3K inhibitors targeting one of the PI3K isoforms have enhanced, precise targeting and decreased toxicity compared with pan-PI3K inhibitors. Isoform-specific PI3K inhibitors may need appropriate patient identification according to sensitivity and resistance markers. In 2014, Idelalisib (a PI3Kδ inhibitor) is approved by the FDA for the treatment of chronic lymphocytic leukemia, relapsed follicular B-cell non-Hodgkin lymphoma, and relapsed small lymphocytic lymphoma (94). In 2018, the FDA approved duvelisib (an isoform-specific inhibitor targeting PI3Kγ and PI3Kδ) based on results from the DUO and DYNAMO for adult patients with relapsed or refractory chronic lymphocytic leukemia or small lymphocytic lymphoma after more than two previous therapies (95, 96). In 2019, the FDA approved alpelisib (a PI3Kα inhibitor) in combination with fulvestrant for postmenopausal women, and men, with hormone receptor-positive, human epidermal growth factor receptor 2 -negative, PIK3CA-mutated, advanced or metastatic breast cancer based on the results from the SOLAR-1 trial (97). According to the results of the CBYL719X2101 trial, alpelisib exhibited an acceptable safety and promising antitumor activity in patients with PIK3CA-mutant malignancies, indicating that selective PI3K inhibitors in conjunction with additional antineoplastic drugs may be effective for the treatment of PIK3CA-mutant malignancies (98). In 2021, the FDA approved umbralisib (a PI3Kδ/CK1ϵ inhibitor) for the treatment of marginal zone lymphoma and follicular lymphoma based on the results from the UTX-TGR-205 trial (99). In addition to idelalisib (δ), alpelisib (α), duvelisib (δ/γ), and umbralisib (δ), other isoform-selective PI3K inhibitors include serabelisib (a PI3Kα inhibitor), GSK2636771 (a PI3Kβ inhibitor), Zandelisib (a PI3Kδ inhibitor), AMG319 (a PI3Kδ inhibitor), linperlisib (a PI3Kδ inhibitor), parsaclisib (a PI3Kδ inhibitor), leniolisib (a PI3Kδ inhibitor), eganelisib (a PI3Kγ inhibitor), tenalisib (a PI3Kδ/γ inhibitor), taselisib (a PI3Kα/δ/γ inhibitor), AZD8186 (a PI3Kβ/δ inhibitor), and AZD8835 (a PI3Kδ/α inhibitor).

### Akt Inhibitors

AKT, an effector of the PI3K/AKT/mTOR pathway to activate tumors, is a promising target. The Akt kinase family comprises the AKT1, AKT2, and AKT3 isoforms. AKT activity is controlled in an Akt-dependent manner *via* phosphorylation and dephosphorylation. Akt inhibitors have been classified into three categories depending on how they impede Akt activity. ATP-competitive inhibitors reduce the phosphorylation of Akt by competing with ATP. Allosteric inhibitors prevent Akt from interacting with its substrate by causing conformational transitions in enzymic structure. Irreversible inhibitors are another less common type of Akt inhibitor. ATP-competitive inhibitors (GSK690693, ipatasertib, uprosertib, and capivasertib) and allosteric inhibitors (MK-2206) have demonstrated the more potent inhibition of Akt in malignant cells. ATP-competitive inhibitors attach to the active conformation of Akt, in which the pleckstrin homology (PH) domain has swung away from the kinase domain and exposed the ATP-binding pocket, thereby blocking the activity of Akt. Allosteric inhibitors can impede the localization of AKT to the plasma membrane and prevent the phosphorylation and activation of AKT.

In the EAY131-Y trial, the objective response rate of single-agent capivasertib (AZD5363) was 28.6% (N=35) in patients with an AKT1 E17K-mutated tumor. One patient with endometrioid endometrial cancer had a complete response and was still on treatment after 35.6 months. Moreover, 46% (N=35) of patients had stable disease, and 6% (N=35) of patients had progressive disease, suggesting that capivasertib shows clinically substantial efficacy in refractory malignant tumors (100). Adding pan-AKT inhibitor capivasertib to docetaxel and prednisolone did not improve a composite progression-free survival in metastatic castration-resistant prostate cancer regardless of whether the PI3K/AKT/PTEN pathway was activated or not in the ProCAID trial (101). After a median follow-up of 4.9 months, median progression-free survival was 10.3 months for patients with metastatic, estrogen-receptor-positive breast cancer treated with capivasertib plus fulvestrant compared with 4.8 months for patients treated with fulvestrant plus placebo in the FAKTION trial (102). The combination of capivasertib and paclitaxel for metastatic triple-negative breast cancer can contribute to significantly prolonged progression-free survival (5.9 months *vs*. 4.2 months) and overall survival (19.1 months *vs*. 12.6 months) compared with paclitaxel plus placebo in the PAKT trial. The results suggested that capivasertib plus paclitaxel has potential as a first-line treatment for metastatic triple-negative breast cancer. In PIK3CA/AKT1/PTEN-altered patients, median progression-free survival with capivasertib plus paclitaxel was 9.3 months while placebo plus paclitaxel was only 3.7 months. Severe adverse events (grade 3-4) were more common in patients treated with capivasertib plus paclitaxel (P < 0.01). The most common adverse events with capivasertib plus paclitaxel versus paclitaxel plus placebo were diarrhea (13% *vs*. 1%), infection (4% *vs*. 1%), neutropenia (3% *vs*. 3%), rash (4% *vs*. 0%), and fatigue (4% *vs*. 0%) (103).

Targeting AKT signaling ipatasertib (GDC-0068) exhibited an acceptable safety profile and significant disease control in patients with AKT-activated solid tumors (104). After a median follow-up of 19 months, the inhibition of PI3K/AKT and androgen-receptor dual pathways by ipatasertib (an ATP-competitive AKT inhibitor) plus abiraterone can improve the median radiographical progression-free survival of metastatic prostate cancer patients with PTEN loss compared with placebo plus abiraterone (18·5 months *vs*. 16·5 months) in the IPATential150 trial. 39% (21/546) of patients treated with the placebo plus abiraterone and 70% (386/551) of patients treated with ipatasertib plus abiraterone experienced grade 3 or higher adverse events. Adverse events led to drug discontinuation in 5% (28/546) of patients treated with the placebo plus abiraterone and 21% (116/551) of patients treated with ipatasertib plus abiraterone (105). In the intention-to-treat population, the combination of ipatasertib and paclitaxel for triple-negative breast cancer can result in significantly prolonged median progression-free survival compared with paclitaxel plus placebo (6.2 months *vs*. 4.9 months) in the LOTUS trial. Median progression-free survival in PTEN-low patients with ipatasertib plus paclitaxel was 6.2 months compared to placebo plus paclitaxel in 3.7 months. Serious adverse events occurred in 28% (17/61) of patients in the ipatasertib plus paclitaxel group and 42% (26/62) of patients in the placebo plus paclitaxel group. The ipatasertib plus paclitaxel group was mainly associated with severe adverse reactions related to infections and gastrointestinal effects, while the placebo plus paclitaxel group was mainly associated with severe adverse reactions related to infections (106).

In the I-SPY 2 trial, MK-2206 (an allosteric inhibitor) combined with standard neoadjuvant chemotherapy contributed to higher pathologic complete response rates in human epidermal growth factor receptor 2 (HER2)-positive, hormone receptor (HR)-negative early-stage breast cancer. Substantial skin adverse reactions are observed, but adverse events, such as rash, can be controlled. Although MK-2206 is not currently being explored further in breast cancer, this type of Akt inhibitor is still promising for clinical use (107).

### mTOR Inhibitors

The mTOR kinase family mainly consists of three functional components: mTOR1, mTOR2, and mTOR3. mTOR1 and mTOR2 are linked with cancer. mTORC1 serves as a downstream effector for several commonly disrupted oncogenic pathways, including the PI3K/AKT and MAPK pathways, and the mTOR pathway is overactive in various tumor types, making mTOR a target for cancer treatment. mTOR inhibitors are a type of drug that works by selectively inhibiting mTOR activity. Generally, mTOR inhibitors are classified into two categories: rapamycin and its analogs (rapalogs), and ATP-competitive mTOR kinase inhibitors. The former is capable of suppressing mTORC1, and the latter can suppress mTORC1/2.

Rapamycin (Sirolimus), a rapalog, was initially used as an immunosuppressant in patients experiencing organ transplantation and it also has anti-proliferative properties. A phase I/II study of pemetrexed in combination with sirolimus in recurrent, metastatic non-small cell lung cancer revealed synergistic benefits when sirolimus was added to pemetrexed (108). In 2007, temsirolimus (CCI-779) was the first rapalog to be approved by the Food and Drug Administration (FDA) for the treatment of advanced renal cell carcinoma (109). FDA approves everolimus (a rapalog) for treatment of various diseases, such as renal cell carcinoma, progressive neuroendocrine tumors of pancreatic origin (PNET), postmenopausal women with advanced hormone receptor-positive, HER2-negative breast cancer, neuroendocrine tumors (NET) of gastrointestinal (GI) or lung origin, tuberous sclerosis complex (TSC)-associated partial-onset seizures, TSC-associated subependymal giant cell astrocytoma (SEGA) and TSC-associated renal angiomyolipoma. In the MIRACLE study, the progression-free survival of patients with HR-positive, ERBB2-negative premenopausal advanced breast cancer treated with everolimus plus letrozole was substantially longer than that of patients treated with letrozole (19.4 months *vs*. 12.9 months; P=0.008), suggesting the effectiveness of everolimus among patients who experienced disease progression and took the same endocrine therapy (110). Compared to fulvestrant plus vistusertib (a dual mTORC1 and mTORC2 inhibitor) or fulvestrant alone, the combination of fulvestrant and everolimus (a mTORC1 inhibitor) resulted in a substantially longer progression-free survival in patients with hormone receptor-positive metastatic breast cancer. Adding vistusertib to fulvestrant failed to show a benefit in the MANTA study (111). In the AcSé-ESMART trial, the CDK4/6 inhibitor ribociclib combined with topotecan and temozolomide (TOTEM) or everolimus was well-tolerated in children with advanced malignancies (112).

The development of resistance to rapamycin analogs seems inevitable due to compensatory activation of the PI3K/Akt pathway. Several drugs have been developed as ATP competitors that block the catalytic activity of mTOR to overcome the ineffectiveness of rapamycin in antitumor therapy. Researchers are now focused on developing mTORC1/2 complex inhibitors such as vistusertib (AZD2014), sapanisertib (TAK-228), AZD8055, and PP242 to overcome this shortcoming of rapamycin analogs (113). 118 postmenopausal women with hormone receptor-positive, human epidermal growth factor receptor 2 negative advanced/metastatic breast cancer participated in a phase IB/II study (NCT02049957) in which the safety, tolerability, and antitumor activity of sapanisertib plus exemestane or fulvestrant were assessed. The combination of sapanisertib with exemestane or fulvestrant has a maximum tolerable dosage of 4 mg once daily. Sapanisertib plus exemestane or fulvestrant showed therapeutic benefit in postmenopausal women with pretreatment everolimus-sensitive or everolimus-resistant breast cancer with clinical benefit rate at 16 weeks (CBR-16) of 45% versus 23% in everolimus-sensitive versus everolimus-resistant subgroups. Molecular analysis revealed a positive correlation between the presence of an AKT1 mutation and improved effectiveness (114). In comparison to the combination of fulvestrant and vistusertib, or to fulvestrant alone, fulvestrant with everolimus showed a substantially longer progression-free survival in patients with hormone receptor-positive metastatic breast cancer. The addition of the dual mTORC1 and mTORC2 inhibitor vistusertib to fulvestrant failed to show any advantage in the MANTA trial (111). Besides, vistusertib was well tolerated in children with advanced malignancies. However, the study arms were discontinued due to the lack of tumor responses and a failure to engage the target (113). Nonetheless, dual mTORC1 and mTORC2 inhibitors targeting the PI3K/AKT/mTOR pathway in the treatment of malignant tumors are still being investigated.

### Dual PI3K/mTOR Inhibitors

Dual PI3K/mTOR inhibitors interact with the ATP-binding cleft of both PI3K and mTOR, reducing the kinase activity of both enzymes and impacting pathway activities more effectively than mTOR kinase inhibitors alone. Dual PI3K/mTOR inhibitors have shown some promise in the early-stage trial. Additional research is required to establish whether dual PI3K/mTOR inhibitors are more effective than mTOR inhibitors. Dual PI3K/mTOR inhibitors such as dactolisib (BEZ235), apitolisib (GDC-0980), gedatolisib (PF-05212384), bimiralisib (PQR309), paxalisib (GDC-0084), and voxtalisib (SAR245409, XL765) have shown substantial anticancer efficacy in various tumor xenografts (9, 115–117).

Treatment with dactolisib (BEZ235), when compared to everolimus, has not been shown to improve effectiveness in patients with advanced pancreatic neuroendocrine tumors who have not previously received mTOR inhibitor treatment, and it may have a worse tolerability profile. The limited effectiveness and poor tolerability of dual PI3K/mTOR inhibitors may restrict their potential for clinical applications (118). In a phase Ib trial (NCT01634061), individuals with castration-resistant prostate cancer were administered dactolisib (BEZ235) plus abiraterone acetate. Eighteen individuals (N=25) were randomized to receive dactolisib plus abiraterone acetate at the first dosage level (200 mg bid) in the dactolisib plus abiraterone acetate arm (NCT01634061). Five dose-limiting toxicities were found in nine individuals. Dactolisib plus abiraterone acetate in castration-resistant prostate cancer will not be studied further due to the available pharmacokinetics, safety, and effectiveness evidence (119). A phase Ib dose-escalation trial (NCT01508104) showed that dactolisib with everolimus was neither efficacious nor tolerable enough in patients with advanced malignancies. Systemic exposure to dactolisib increased in a dose-proportional manner, whereas oral bioavailability was poor, perhaps due to gastrointestinal toxicity (120). Additionally, the combination of gedatolisib (dual PI3K/mTOR inhibitor) with carboplatin and paclitaxel was tolerated in patients with advanced solid tumors, and preliminary effectiveness was seen particularly in clear cell ovarian cancer in a phase I dose-escalation trial (121). In a phase II study (MAGGIE), the efficacy of apitolisib (a dual PI3K/mTOR inhibitor) was assessed in patients with advanced endometrial cancer. The anticancer efficacy observed with apitolisib was restricted by its tolerability. A comprehensive molecular profile revealed that 57% (N=46) of patients had at least one PIK3CA, PTEN, or AKT1 mutation. Each of the three individuals who had a confirmed response had at least one PI3K pathway gene mutation, indicating that patients with mutations in the PI3K pathway may have benefited more with apitolisib. The most common grade 3 or higher adverse events were rash (maculopapular, acneiform). Two serious adverse events due to grade 3 rash were associated with MK-2206 (122). In addition, enrichment for PI3K pathway biomarkers may be beneficial for future research of more selective inhibitors in PI3K/mTOR signaling.

## Conclusion and Prospect

Multiple studies have revealed significant genetic alterations in cancer cases. Due to the heterogeneity and complexity of tumors, the mechanism of carcinogenesis remains undetermined. Targeted treatment is developed as an evolving strategy to improve the survival of cancer patients. The PI3K/AKT/mTOR pathway is the most commonly disrupted in cancer. This hyperactive pathway offers possibilities and opportunities for drug research and discovery. The previous studies have exhibited that significant PI3K/AKT/mTOR axis is significantly altered in cancer and targeting this axis with multiple inhibitors can modulate a variety of cellular processes such as cell proliferation, autophagy, apoptosis, angiogenesis, EMT, and chemoresistance. Pharmaceutical research has contributed to the development of various types of inhibitors that target distinct components of this axis. mTOR inhibitors, PI3K inhibitors, Akt inhibitors, and dual PI3K/mTOR inhibitors have all been studied as monotherapy or in combination with other inhibitors in the treatment and prevention of cancer. Despite substantial advancements, effective management of cancer remains a challenge due to the heterogeneity of cancer and proper patient identification for targeted therapy.

There are still many issues in this review that deserve attention or need to be further explored. For example, most of the data on precision medicine come from developed countries in Europe and the United States, while data from other regions, such as Asia, are relatively rare. Differences in data on precision medicine from different populations need to be explored more widely. Besides, multiple TCGA datasets included more primary tumors, and the genetic profiles of primary tumors may differ from those of advanced or metastatic tumors. Moreover, combination with PARP inhibitors or immune checkpoint inhibitors is a promising direction that needs further exploration. Additionally, multiple combinations of targeted therapy strategies are appropriate only for specific cancer types. For example, dactolisib plus abiraterone acetate (a CYP17 inhibitor) is mainly used to treat castration-resistant prostate cancer. Capivasertib plus fulvestrant (an estrogen receptor antagonist) is primarily used to treat metastatic, estrogen-receptor-positive breast cancer. Furthermore, PI3K/AKT/mTOR inhibitor resistance and its mechanism need to be further elucidated.

Targeted therapy targeting the PI3K/AKT/mTOR pathway may produce a variety of adverse reactions and is prone to progress due to drug resistance. Tumor-specific research should be a severe issue, and appropriate dosing regimens need to be explored to make PI3K/AKT/mTOR inhibitors more tolerable and efficient. Increasing the number of clinical studies is an effective way to tailor treatments for cancer patients. Further research is needed to uncover the resistance mechanisms of PI3K/AKT/mTOR inhibitors, explore how to overcome resistance to PI3K/AKT/mTOR inhibitors, and develop new, more rational therapeutic combinations.

## Author Contributions

YP, YW, CZ, WM, and CCZ designed the study and supervised. YP, CZ, and YW collected data. YP performed statistical analysis. YP, WM, and YW interpreted data and drafted the manuscript. CCZ contributed to administrative and technical and material support. All authors contributed to the article and approved the submitted version.

## Funding

This work was supported by the National Natural Science Foundation of China (81660755); and the Science and Technology Project of Shenzhen of China (JCYJ20170307160524377 and JCYJ20190808162605484).

## Conflict of Interest

The authors declare that the research was conducted in the absence of any commercial or financial relationships that could be construed as a potential conflict of interest.

## Publisher’s Note

All claims expressed in this article are solely those of the authors and do not necessarily represent those of their affiliated organizations, or those of the publisher, the editors and the reviewers. Any product that may be evaluated in this article, or claim that may be made by its manufacturer, is not guaranteed or endorsed by the publisher.
